# In vivo evidence of functional and anatomical stripe-based subdivisions in human V2 and V3

**DOI:** 10.1038/s41598-017-00634-6

**Published:** 2017-04-07

**Authors:** Serge O. Dumoulin, Ben M. Harvey, Alessio Fracasso, Wietske Zuiderbaan, Peter R. Luijten, Brian A. Wandell, Natalia Petridou

**Affiliations:** 1 Department of Experimental Psychology, Helmholtz Institute, Utrecht University, Utrecht, Netherlands; 2 Spinoza Centre for Neuroimaging, Amsterdam, Netherlands; 3 Department of Radiology, University Medical Centre, Utrecht, Netherlands; 4 Department of Psychology, Stanford University, California, USA; 5 Brain Center Rudolf Magnus, University Medical Centre Utrecht, Utrecht, Netherlands

## Abstract

Visual cortex contains a hierarchy of visual areas. The earliest cortical area (V1) contains neurons responding to colour, form and motion. Later areas specialize on processing of specific features. The second visual area (V2) in non-human primates contains a stripe-based anatomical organization, initially defined using cytochrome-oxidase staining of post-mortem tissue. Neurons in these stripes have been proposed to serve distinct functional specializations, e.g. processing of color, form and motion. These stripes represent an intermediate stage in visual hierarchy and serve a key role in the increasing functional specialization of visual areas. Using sub-millimeter high-field functional and anatomical MRI (7T), we provide *in vivo* evidence for stripe-based subdivisions in humans. Using functional MRI, we contrasted responses elicited by stimuli alternating at slow and fast temporal frequencies. We revealed stripe-based subdivisions in V2 ending at the V1/V2 border. The human stripes reach into V3. Using anatomical MRI optimized for myelin contrast within gray matter, we also observe a stripe pattern. Stripe subdivisions preferentially responding to fast temporal frequencies are more myelinated. As such, functional and anatomical measures provide independent and converging evidence for functional organization into striped-based subdivisions in human V2 and V3.

## Introduction

Visual cortex is functionally and structurally organized at multiple spatial scales. One important spatial scale is the retinotopic map^1^. The occipital lobe is tiled by a set of such maps, each spanning several square centimeters. A second, larger scale, is defined by clusters of adjacent maps that share a common fovea-periphery organization but mirror-reversing angular representations^2^. A third organizational scale is present within individual maps. Perhaps the best known is the interleaved ocular dominance columns within the primary visual cortex (V1), which comprise an orderly pattern at the millimeter scale. The existence of an organizational pattern is most convincing when the same organization can be detected using a combination of functional and structural methods.

Invasive studies in non-human primates established a millimeter scale organization in the V2 field map. This structure is identified by post-mortem staining of tissue for cytochrome oxidase (CO)^3–5^; which in most cases also stain for myelin^4, 6, 7^. The CO staining pattern in macaque comprises three types of stripes that begin at the V1/V2 border and extend across V2 and perhaps into V3. The three types of stripes are distinguished by the density and size of the CO stain: “thick”, “thin” and “pale”.

Primary visual cortex (V1) can also be partitioned into two regions by the pattern of CO staining: regions that stain heavily (patches) and the regions between (interpatches). Non-human primate studies propose that the thin stripes in V2 receive V1 projections principally from V1 patches^8^; thick and pale strips receive a similar laminar pattern of V1 inputs principally from the interpatches. The most significant difference between the inputs to the thick and pale stripes is a larger projection from layer 4B to thick stripes, which in turn predominantly takes its inputs from magnocellular layers of the LGN. V2 also receives a significant thalamic input from the pulvinar^9^, and the density of these inputs is complementary to the V1 inputs.

Physiological measurements in non-human primate show that functional responses differ between stripes^10–14^. For example, maps of motion direction tuning are found in the thick and pale stripes, but not in the thin stripes^6^. Furthermore, color and disparity responses may also differ across stripes^15, 16^.

There is also histological evidence of a stripe-based organization in human V2^17–20^. The human stripes can be seen in CO and myelin staining. There is a suggestion that the CO staining may be more “patchy” rather than stripe-like, and it is unclear whether human V2 supports the thin-thick stripe distinction^20^.

The organization within human V3 is also unclear. Similar stripe-like compartmental organizations may exist in V3. Yet only a handful of studies in other non-human primates suggest a compartmental organization in V3 resembling V2-stripes^21–24^.

A second difficulty with human V3 organization is that there has been a debate about the existence of V3 across primates^21^. Although this debate appears to be resolved^25^, there remain questions about the homology between V3 in different species. In particular, V3/V3A motion-sensitivities are reversed between humans and macaques^26, 27^, questioning extrapolation from non-human primates to humans. In part because of these uncertainties, human histology studies have delineated V2 based on the termination of the stripes^17, 20^, consequently assuming no stripe structures in V3. This assumption may prove to be false and is open to study.

The advent of functional magnetic resonance imaging (fMRI) has allowed the delineation of human cortical organization at increasingly higher resolutions *in vivo*, again ranging from visual field maps^1^ to columnar structures^28–31^. The challenges in measuring these organizations using fMRI vary. Visual field mapping is a standard capability. Reliable columnar reconstruction, on the other hand, is achieved by only few laboratories due to limitations in sensitivity of typical MRI techniques to the functional signatures of these structures. The difficulties in obtaining *in vivo* evidence of stripe subdivisions in humans arise from similar technical limitations but some evidence exists^32^. In some cases, fMRI data can be guided by our understanding of non-human primates. But, in this case the homologies are not firmly established. Although V1 and V2 seem evolutionary preserved^33^, differences between human and non-human primates make *in vivo* measurements of these functions in humans essential.

Using high-field sub-millimeter resolution MRI (7T), we provide *in vivo* evidence of stripe-based subdivisions in human V2 and V3. The stripe-based subdivisions are found using both functional and anatomical MRI, the latter optimized for myelin contrast within cortex^34^. The functional and anatomical measures correlate within individual subjects, providing independent and converging evidence. These measurements show that neurons with different temporal sensitivity, similar to the temporal frequency specialization that distinguishes parvo- and magnocellular pathways, are segregated in spatial subdivisions in V2 and V3. The high temporal frequency sensitivity arises in the subdivisions containing more myelin.

## Results

### Stripe-based fMRI responses in V2/V3 but not in V1 and V3A

We recorded fMRI responses elicited by viewing concentric gratings whose contrast reversed at a slow (1.5 Hz) or fast (7.5 Hz) frequency. We calculated the phase-specified coherence of each fMRI time series, which is a measure of the amplitude of the fMRI response at the stimulus frequency and phase, adjusted for the hemodynamic delay. The values ranged between −100 and 100%; positive values reflect stronger responses to the slow stimulus and negative values reflect stronger responses to the fast stimulus.

**Figure 1:**
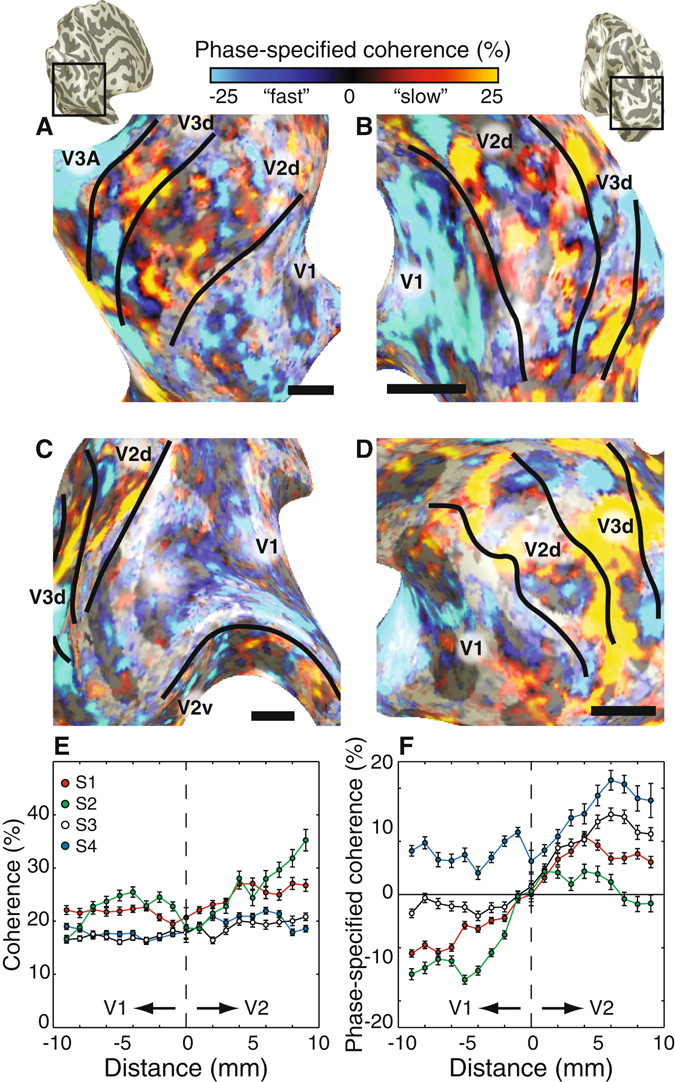
Functional delineation of V2 and V3 stripe-based subdivisions. Phase-specified coherence responses displayed on the left (**A,C**) and right (**B,D**) inflated cortical surfaces of three subjects (panels A,B: S3, panel C: S1; panel D: S2). The panels in A–D show the enlarged view of the dorsal occipital lobe with the identified V1/V2d/V3d/V3A borders (top insets). The color bar indicates the phase-specified coherence values, indicating responses dominated by “fast” (7.5 Hz) or “slow” (1.5 Hz) temporal frequencies. Only phase-specified coherence values exceeding an absolute value of 15% are shown. The black horizontal line indicates 1 cm in each panel. The coherence and phase-specified coherence across the V1/V2 border is shown in panels E and F respectively. The coherence is a measure for overall response modulation irrespective of the relative response preference. The coherence values are similar across the border indicating a similar overall response to the stimulus (**E**). The phase-specified coherence also takes into account which temporal frequency drives the response. The phase-specified coherence changes across the V1/V2 border (**F**) demonstrating a distinct and systematic change in response properties along the V1/V2 border for all four subjects. The error bars reflect standard error of the mean.

The phase-specified coherence values are shown in Fig. 1A–D. The field of view covers V1, V2d, and V3d. It also covered V3A (panel A) and V2v (panel C) in certain cases. As expected, V3A responds stronger to faster temporal frequencies. This is expected based on observations that human -but not macaque- V3A has response properties remarkably similar to MT and presumably also receives a dominant input from the magnocellular stream^26^. V1 also responds stronger to faster temporal frequencies but to a lesser extent (lower phase-specified coherence values). Almost all visual information passes through V1 and we propose that this V1 response may be a consequence of the exact stimulus parameters rather than reflecting a stronger magnocellular input.

Importantly, V1 responds more to the fast stimulus throughout its cortical extent. This pattern changes abruptly at the V2 border. In both V2 and V3 responses vary systematically: within these two maps there is an interleaved series of stripes that respond preferentially to slow or fast temporal frequencies. The spatial organization of these responses is consistent across measurements separated by several days. Based on visual inspection, these sometimes line up into stripes orthogonal to the V1/V2 border and end at the V1/V2 border.

**Figure 2:**
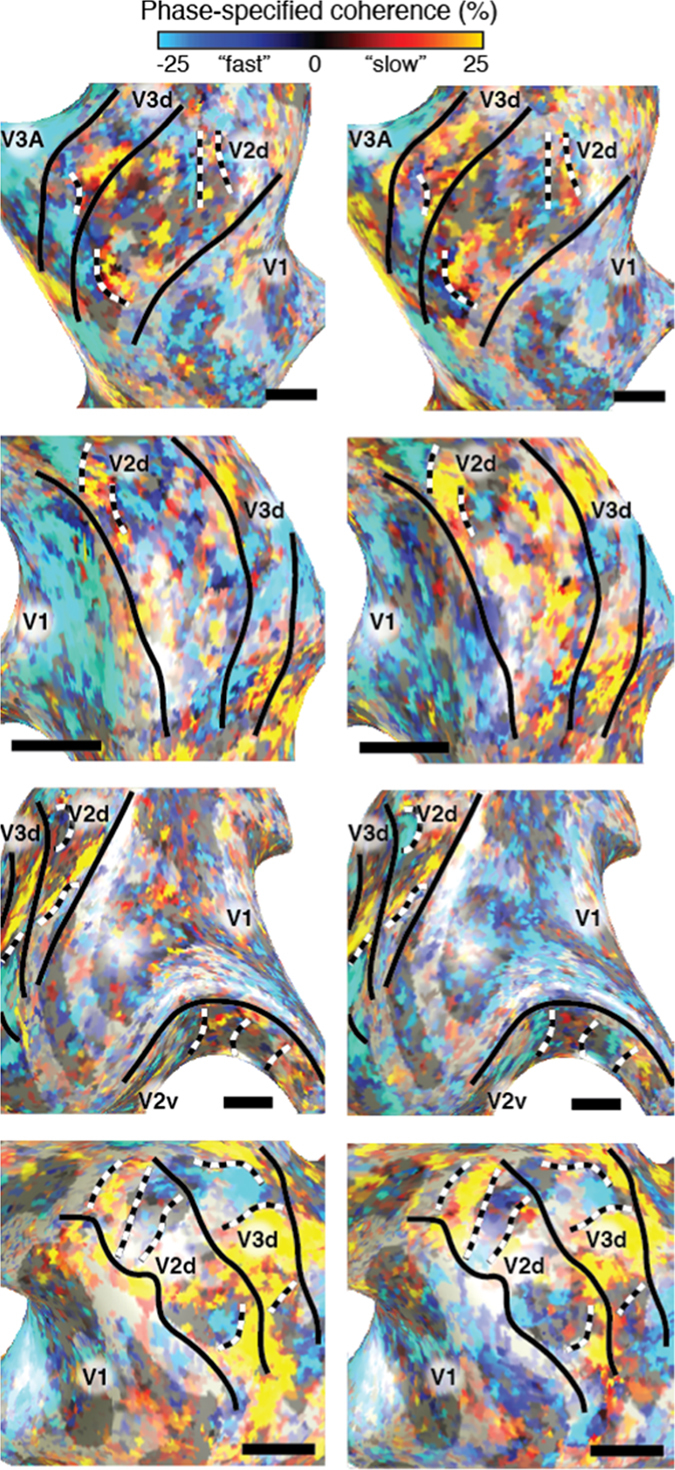
Independent functional delineation of V2 and V3 stripe-based subdivisions. Phase-specified coherence responses displayed on inflated cortical surfaces of the same subjects and in an identical fashion of Fig. 1 (left = odd runs and right = even runs). There is a significant correlation between these independent datasets. Black and white dashed lines illustrate correspondence between odd and even runs.

We quantified the stability of these maps by computing a Spearman correlation coefficient between two independent halves of the data, i.e. odd/even runs and datasets collected on different days (Fig. 2). The mean (and standard error) of the correlation coefficients for V1, V2 and V3 were 0.24(0.04), 0.41(0.04) and 0.55(0.05), respectively. All of these correlations are significant even in the individual comparisons (p ≪ 0.001), indicating that these measurements are reproducible across independent datasets in individual subjects. Correlation coefficients were significantly higher when comparing odd/even runs compared to those acquired on different days (Analysis of Variance). Furthermore, the correlation coefficient is significantly different between visual field maps.

V2 stripes are known to end at the V1/V2 border. To quantify whether there is a signal change at the V1/V2 border, we plotted all V1 and V2 phase-coherence values according to their distance along the cortical surface from the V1/V2 border (up to a maximum distance of 10 mm, Fig. 1E,F). The curve for each subject shows similar coherence to both temporal frequencies (coherence, Fig. 1E). But, for each subject there is a substantial shift in phase-specified coherence indicating a systematic shift in frequency preference when crossing the border between V1 and V2 (Fig. 1F). V1 responds at higher amplitude to faster temporal frequencies. This preference switches in V2. This representation collapses all recording sites in V2 (and consequently all stripes) depending on the distance to V1. Therefore the overall V2 preference depends on the relative weights of the different stripe-types as well as variations in the individual subject’s response bias to each temporal frequency. However in every subject there is a distinct shift of the response profiles in the same direction. These results do not quantify the stripes in V2, but highlight distinct response properties between V1 and V2 which is indicative of the ending of stripe-based patterns at the V1/V2 border. To measure the stripe-based periodicity, we measured the FWHM of the absolute difference between the phase-specified coherence of a given voxel and the neighboring voxels up to 5 mm (radius, geodesic distance). The median (and 95% CI) of the stripe-based subdivisions (FWHM) were in V1: 3.43 [3.19 3.69], V2: 4.54 [4.39 4.70] and V3: 4.61 [4.46 4.78] in millimeters. FWHM in V1 is systematically smaller than in V2 and V3.

### Myelin-contrast measurements in V2 and V3 align with functional variations

**Figure 3:**
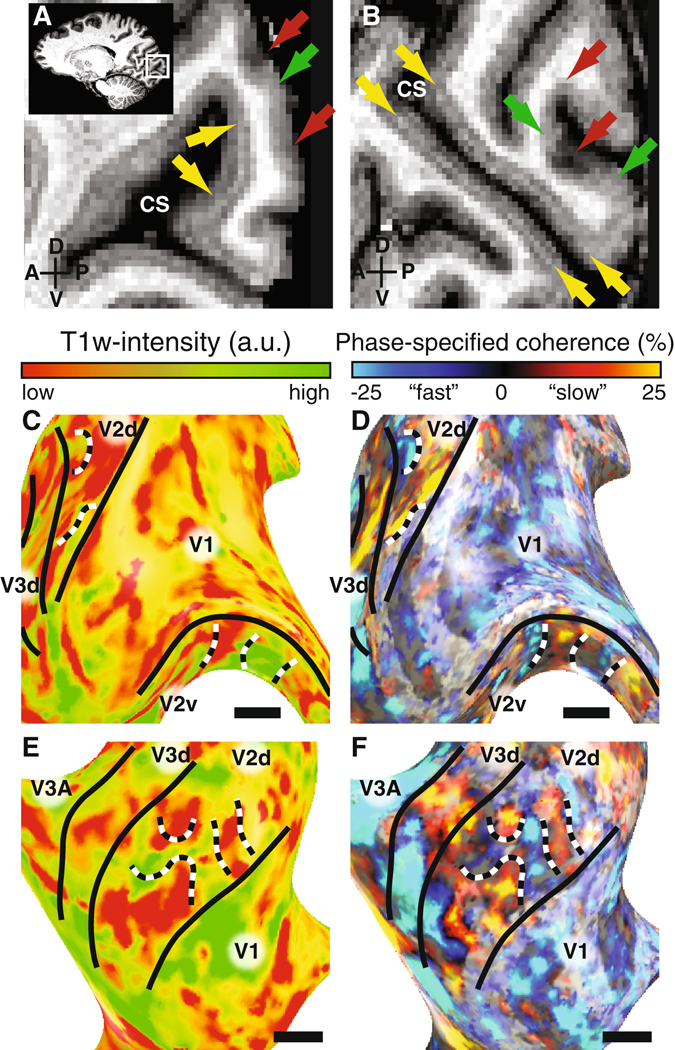
T1w anatomical delineation of V2 and V3 stripe-based subdivisions. High-resolution T1w data is shown in panels A (S1) and B (S2). The data reveal the stria of Gennari (yellow arrows), and stripes in V2 cortex with low and high T1w intensity, associated with less and more myelinated regions (red and green arrows, respectively). The median T1w intensities across the cortical thickness are shown on the cortical surface in panels C (S1) and E (S3). The corresponding functional responses of these subjects are shown in panels D and F (identical to Fig. 1). The black horizontal line indicates 1 cm in each panel. In line with the functional responses a patchy distribution of T1w intensity is observed along visual cortex. Black and white dashed lines mark borders within V2 that illustrate correspondence between T1w intensity changes and functional responses.

We acquired high-resolution T1-weighted (T1w) MR images (500 micron isotropic) optimized for myelin contrast within cortical tissue (Fig. 3)^34^. In V1, these images reveal the stria of Gennari, a band of heavily myelinated tissue in layer 4 that is coterminous with V1 (Fig. 3A,B).

The regional variations in V2 do not appear to be localized to a specific cortical layer, but rather appear more diffuse across layers. These variations were projected on the cortical surface by taking the median value across the cortical layers (Fig. 3C,E). Within V2, V3 but also in V1, local variations in T1w intensity associated with myelin content are apparent.

We quantified the stability of these T1w intensity variations by computing the Spearman correlation coefficient across the data-points within a visual field map between two independent halves of the data, i.e. odd/even runs and two datasets collected on different days. The mean (and standard error) of the correlation coefficients for V1, V2 and V3 were 0.67(0.05), 0.76(0.06) and 0.74(0.06), respectively (average of 4 subjects). All of these correlations are significant even in the individual subjects (p ≪ 0.001). These results indicate that these measurements are repeatable across independent datasets in individual subjects. Correlation coefficients were significantly higher when comparing odd/even runs compared to those acquired on different days (Analysis of Variance). There were no significant differences between visual field maps.

**Figure 4:**
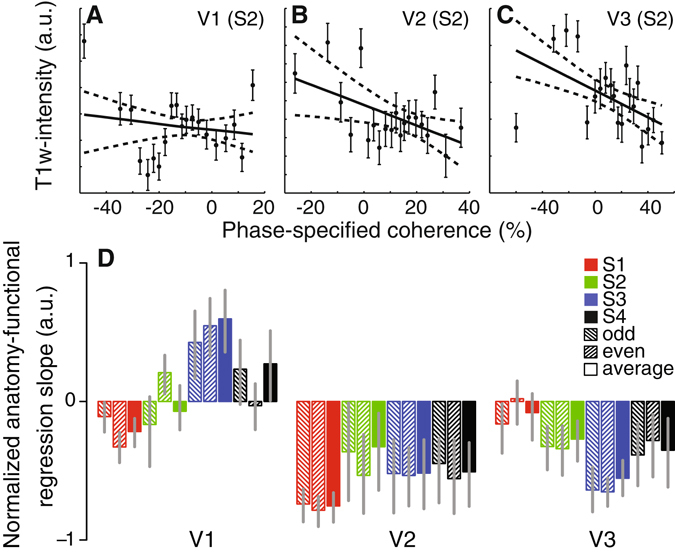
Relation between anatomical and functional variations of V2 and V3 stripe-based subdivisions. The relationship between functional and anatomical variations for subject S2 in V1, V2 and V3 are shown in panels A–C respectively. The solid and dashed lines indicate the linear relationship and 95% confidence interval obtained by bootstrapping the phase-specified coherence quantiles. The normalized slope between functional and anatomical data for all subjects and independent halves of the data are shown in panel D. All error bars reflect the 95% confidence intervals. We observed a significant relationship between T1w intensity and fMRI response in each subject, except S1 V3, providing converging evidence for the V2 and V3 stripe-based subdivisions. We propose that the V2 and V3 subdivisions that respond more to faster temporal frequencies are more myelinated.

The myelin variations resemble the functional stripe-based subdivisions in V2 and V3 (Fig. 2). To quantify this observation we correlated the anatomical and functional data (Fig. 4A–C). Both data were collapsed over cortical layers by taking the median value across layers. In V2 and V3 these correlations were significant and present for all four subjects with the exception of V3 in S1 (Fig. 4D). These correlations were also significant for odd and even runs of the anatomical data with the functional data.

In sum, subdivisions in V2 and V3 that are more responsive to faster temporal frequencies have larger T1w values consistent with an increase in myelin density^35^. We interpret this as evidence that the stripe-based subdivisions that are more responsive to high temporal frequency stimuli also contain more myelin.

To measure the stripe-based periodicity, we measured the FWHM of the absolute difference between the de-curved T1w signal of a given voxel and the neighbouring voxels up to 5 mm (radius, geodesic distance). The median (and 95% CI) of the stripe-based subdivisions (FWHM) were in V1: 2.74 [2.48 3.04], V2: 3.40 [3.28 3.52] and V3: 3.40 [3.19 3.62] in millimetres. FWHM in V1 is systematically smaller than in V2 and V3.

T1w intensity variations are also evident in V1. This is likely caused by including all layers in the projection to the cortical surface, which reduces the sensitivity to the stria of Gennari in layer 4. This is unlike previous reports where similar visualization focused on the middle of the cortex^35–39^ and included spatial smoothing to improve the signal-to-noise. Although V1 T1w intensity variations in V1 are significantly correlated with functional variations in some subjects (S1 and S3), there is no consistent relationship across subjects.

## Discussion

We provide *in vivo* evidence for stripe-based subdivisions in human V2 and V3. The evidence is based on both variations in functional responses and T1w intensity variations associated with myelin content. The functional response profile changes abruptly across the V1/V2 border, highlighting the difference between V1 and V2. Furthermore, these variations in functional responses and myelin are spatially correlated, providing independent confirmation and converging evidence of the V2 and V3 stripe-based subdivisions. Stripe-based subdivisions represent an intermediate stage between intermingling of neurons with different functional specializations and their separation into distinct, functionally specialized cortical areas. Extension of this process into V3 may represent a further stage of a similar process.

The homology between these V2 subdivisions and previous reports of V2 stripes is not certain. First, we describe two subdivisions in V2, whereas most previous studies distinguish at least three types of stripes^3–7^. We propose that our stripe-based subdivisions likely collapse two stripe types and speculate that our subdivisions distinguish ‘thick’ versus both ‘thin’ and ‘pale’ stripes. Our proposal is based on previous non-human histological observations suggesting that ‘thick’ stripes in V2 receive a larger projection from layer 4B in V1 which is dominated by magnocellular input^10–14^. Furthermore, the stripe-based spatial periodicity of ~6 mm (full cycle) corresponds closely to estimates based on human histology, i.e. 4.6 mm^20^, 7 mm^17^, and 8 mm^18, 19^. In the case where human histology could not distinguish thick versus thin stripe types^20^, we doubled their reported “thick/thin-pale” periodicity assuming a “thick-pale-thin-pale” cycle. Taken together these different measurements suggest an existence of three types of stripes in humans, though methodological factors sometimes cannot dissociate between all stripe types. Therefore we believe that our functional results are in line with previous V2 stripe observations.

The subdivisions that we observed in V2 and V3 appear more patchy than stripy. Typically, V2 stripes appear stripy in non-human CO histology but also in some human histology using different stains (for example for myelin). However, patchy CO patterns have also been observed in non-human primate^40^ and human^17^ V2. The patchy versus stripy distinction may reflect a genuine difference between individuals, but can also be attributed to methodological limitations both in previous histological studies and our *in vivo* study. In our study, there are various methodological factors that could make biological stripe divisions appear patchy, such as errors in surface reconstruction, partial volume effects, and variations in local sensitivity in functional and anatomical imaging. Regardless, our data support a stripe-based dissociation within V2 *in vivo* and in humans.

A recently published study using *in-vivo* high resolution imaging explored the stripe organization in areas V2 and V3 in humans^32^. Nasr, *et al*.^32^ found evidence for color-selective and disparity selective sensitivity stripes in human V2 and V3. On the other hand, the visual manipulation we adopted here implies a stripe subdivision based on temporal frequency sensitivity and anatomy. Taken together, the present study and the study of Nasr *et al*., provide complementary evidence of the stripe organization V2 and V3, in humans. A key difference in the approach between the two studies is that we opted for an independent confirmation of the presence of stripes based on the anatomical evidence provided by the high-resolution T1w scans. Our study is the first to identify column-based (stripes) based on anatomical criteria, *in vivo*, in humans, matching structural and functional stripe organization.

We extend previous observations by measuring both functional and anatomical measures within the same subject. We find that stripe-based subdivisions responding stronger to faster temporal frequencies are also more myelinated. This direct *in vivo* comparison allows for critical insight in contradictory histological evidence. Histological studies have suggested more myelination for thin *or* thick stripes^17^, or (every other) pale stripe^41^. Furthermore, these results may depend on the staining technique^42^. We do not have access to CO-information in our *in vivo* study, and histological studies do not have access to functional responses. However, our finding is consistent with cortical area MT which has similar functional responses^43, 44^ and is also more myelinated as confirmed by both postmortem^7, 17, 18^ and *in vivo* ^36, 38^ observations. Thick stripes project to MT^11^ and, consequently, we propose that the thick stripes are more myelinated.

In V3d, we observe similar stripe-based subdivisions in both functional responses and anatomical measurements. However, the V3 stripe-based subdivisions appear larger than in V2. This may have a biological origin, i.e. the stripe-based subdivisions may be organized according to different principles in V3. Different V3 stripe-based subdivisions should be interpreted with caution; we used a surface-coil for functional imaging and on average V3 was further removed from the coil which can reduce the sensitivity in that region. Notwithstanding, the functional and anatomical correlations were in the same direction as V2.

Our observation of stripe-based subdivisions in V3 extends previous non-human and human observations. Previously, only a handful of non-human primate studies have suggested stripe-based structures in V3^21–23^. In part due to the sparse non-human primate data, human histological approaches have assumed that stripe-based structures end at the V2/V3 border^17, 20^. Consequently, when defining V2 based on stripe-structures in humans, V2 was found to be larger than V1^20^. This contrasts with non-human primates^11^, other human histological criteria^45^ and human neuroimaging^46^. Based on visual field map criteria, our results suggest that defining V2 size based on stripe-appearance in humans would combine V2 and V3, which can explain the large V2 sizes in the previous reported measurements. This finding is in line with a hypothesis of Tootell *et al*.^47^, who tried to reconcile their earlier histological data^17^ with fMRI visual field mapping results (Fig. 1 and Fig. 13, in the respective publications). In short, in line with Nasr, *et al*.^32^ we propose that the V3 stripe-based subdivisions exist in humans and likely across primates.

V1 responses were more homogeneous than V2. Overall V1 responses were stronger to the faster temporal frequency. Because almost all visual information passes through V1 we do not interpret this as a bias towards faster temporal frequencies, but as a consequence of the exact stimulus parameters. We suspect that the exact choice of spatial frequency, contrast and temporal frequencies could bias the overall V1 response.

Sun *et al*.^31^ dissociated a temporal-frequency dependent columnar architecture using a similar paradigm in V1 but did not dissociate the V2 stripe-based subdivisions. Our data, on the other hand, dissociates V2 stripe-based subdivisions but we were not able to reconstruct temporal-frequency dependent columns in V1. These differences are not inconsistent and can be explained by methodological factors. We applied a high isotropic resolution, optimized for cortical surface visualization. The cortical surface representation collapses over layers and sacrifices spatial resolution, but can be applied throughout the cortex. Sun and colleagues, on the other hand, optimized the in-plane resolution of a slice placed on a flat piece of V1 cortex. This is optimal to visualize the V1 columns in a local piece of cortex but not beyond that piece of cortex in V1 or in V2. In our data, we do observe some local variations in the fMRI response in V1. We suspect that these are aliased or low-pass versions of the columnar structure, which would be consistent with the observations of Sun *et al*.^31^.

In the few cases we obtained data from V3A, we observed a strong response to faster temporal frequencies. This is entirely consistent with previous observations of human – but not macaque – V3A sensitivities^26, 27^.

Differences in myelin distribution can dissociate cortical areas. Recent advances in MRI methodology have revealed myelin variations *in vivo* in human visual cortex that is consistent with histological and functional definitions^34–39^. The correlation of T1 intensity with myelin content has been shown in humans post-mortem combining MR imaging and histology^34, 48, 49^ and in non-human primates combining *in vivo* T1 measurements with post-mortem myelin staining in the same animals^35^. In line with previous observations, we find intensity variations associated with myelin content that closely match the known myelin distribution in visual cortex, for instance the stria of Gennari. Previous MRI studies in humans did not highlight differences within a cortical region, which may be a consequence of smoothing of the data and averaging of individual subjects. With the analysis approach here we find myelin variations within a cortical visual field map and show that these local variations correlate with variations in functional responses within individuals.

We report data from four subjects. In all four, we found consistent stripe-based response variations V2 and V3 that correlated with variations in myelin distribution. This demonstrates the ability to reconstruct stripe-based structures in humans and *in vivo*. There are many factors that could impede successful reconstruction of these stripe-based structures, including subject-motion, functional-anatomical alignment, and spatial specificity of the fMRI. Besides technical limitations, biological variations in individual subjects may limit the ability to visualize stripe-based structures. For example, our study demonstrates differences in response biases towards the fast and slow temporal frequencies, and differences in stripe-based structures sizes. Most visual structures vary with a factor of two or more between subjects and this natural variation may push certain structures below the resolution limit in certain subjects.

In short, we demonstrate stripe-based subdivisions in both V2 and V3 based on functional and anatomical properties. These measurements provide evidence for the notion that neurons in V2 and V3 are segregated into distinct functional subdivisions, and that these subdivisions are associated with different degrees of myelination. They further provide evidence that *in vivo* human data can dissociate functional and anatomical subdivisions at high spatial resolutions.

## Methods

### Subjects

Measurements were obtained from four subjects (one female; age range: 28–39). All subjects had normal or corrected-to-normal visual acuity. All experimental procedures were conducted in accordance with the 1964 Declaration of Helsinki (most recently amended in 2008, Seoul), performed with the informed written consent of the subjects and approved by the Human Ethics Committee of the University Medical Center Utrecht.

### Stimuli

The visual stimuli were presented on a back-projection screen and viewed using prisms and an angled mirror within the bore of the MR scanner. The stimuli were generated using the Matlab programming environment using the PsychToolbox^50, 51^ on a MacBook Pro.

Two visual stimuli were presented that consisted of a concentric circular grating of 20% contrast with a spatial frequency of one cycle per degree (visual field of view: 11 degrees diameter). The contrast polarity reversed at a temporal frequency of either 1.5 or 7.5 Hz. These two stimuli alternated in a block design (13s/block) and were chosen to elicit differential responses in parvo- versus magnocellular dominated pathways respectively^10^. Though there may be considerable mixing of both pathways in each stripe-type^52^, we assume there is enough of a bias in the mixture to reveal the stripes. Retinotopic scans, i.e. rotating wedge stimuli, were also acquired in both sessions to delineate the visual field maps. The parameters of rotating wedge stimuli are described in previous studies^1^. During both scans, subjects fixated a dot in the centre of the display, and responded when this changed colour.

### Magnetic resonance imaging

fMRI data were acquired with a 7T Philips Achieva scanner using a volume transmit (Nova Medical, MA, USA) and a 16-channel receive surface-coil^53^. Subjects’ heads were positioned with tight foam padding that minimized head motion. The functional data were acquired using a 3-dimensional segmented gradient-echo echo-planar-imaging (EPI) sequence with 29 coronal slices and acquisition time of 2.6 s per volume (TR/TE 35/25 ms, flip angle 20 degrees, SENSE factor 3.5 in the right-left direction, echo planar factor: 17, bandwidth (in the phase-encode direction): 59 Hz/pixel (with potential blurring in the phase-encode direction estimated at ~2%^54^, voxel size = 0.9 × 0.9 × 1.0 mm, FOV = 120 (right-left) × 120 (feet-head) × 29 (anterior-posterior) mm^3^, 80 time-frames, scan duration about 4 min). Each subject participated in at least two fMRI sessions, and each session comprised between 4 and 7 scans.

In a separate session, high-resolution T1-weighted (T1w)^34^ anatomical MRI images were acquired with the 7T scanner and a 32-channel head coil (Nova Medical, MA, USA). The images were obtained with a 3-dimensional MPRAGE sequence adjusted to obtain a strong myelin contrast in gray matter based on the observation that in high myelin regions T1 is approximately 15% shorter at 7T^34, 35^. A strong contrast in high myelin content gray matter was obtained by setting the time delay (TD) between inversion pulses such that the difference in longitudinal magnetization prior to inversion pulse was decreased between white and gray matter, and by setting the inversion delay (TI) such that the gray matter signal was just above the null point. An optimum contrast is obtained at TD = 6 s and TI = 1200 ms. Other sequence parameters were: TR/TE 8/3 ms, flip angle: 8 degrees, voxel size = 0.5 mm isotropic, FOV: 140 × 140 × 30 mm, 60 coronal slices, bandwidth 202 Hz/pixel, turbo factor: 275, adiabatic inversion, and no acceleration. The imaging volume was at about the same location in visual cortex as the functional images.

At this TI and TD, CSF appears bright in magnitude images. Therefore images from the real component of the complex MR signal were also reconstructed, in which CSF is dark, and were used to mask CSF in the magnitude images by intensity thresholding. The resulting magnitude images were used in subsequent analysis steps.

### Functional data-analysis

Data were analyzed using the open-source mrVista software (http://vistalab.stanford.edu/vistawiki). The first 13 time frames in each functional run were discarded due to start-up magnetization transients in the data. The remaining time-frames were corrected for motion, aligned and interpolated to the anatomical data^55^. Baseline drifts of the fMRI time series were removed by high-pass temporal filtering. fMRI data were analyzed in the Fourier domain and we computed the coherence and phase-specified coherence per voxel. The coherence of each fMRI series at the fundamental stimulus frequency is a measure of the strength of the BOLD response^56^ but does not specify to which stimulus. The phase-specified coherence measures the amplitude of the BOLD response and further distinguishes between the two stimulus types.

The coherence (*C*) was computed as:

1 $$C=100\cdot \frac{{A}_{0}}{\sqrt{\sum {A}_{f}^{2}}},$$

where *A*_*f*_ are the amplitudes of each Fourier component (*f*), and *A*_*0*_ is the amplitude at that stimulation frequency. The coherence values vary between 0% and 100%, larger values indicate stronger responses to the stimulus manipulation. Data were thresholded at a coherence value set at 15% quantile of each single participant coherence distribution (which corresponded to the following coherence values: 13%, 13%, 16% and 15% coherence for subjects 1 to 4, respectively), in order to include voxels responsive to the stimulus manipulation independent of their response phase.

The phase-specified coherence (*C*_*φ*_) was computed as:

2 $${C}_{\phi }=C\cdot \cos \,({\phi }_{0}-{\phi }_{hrf}),$$

where *C* is the coherence (formula 1), *φ*_*0*_ is the phase of the signal at the stimulation frequency, and *φ*_*hrf*_ is the hemodynamic response delay. The hemodynamic delay (*φ*_*hrf*_) was estimated based on the response profiles of the data (see below). The phase-specified coherence combines the coherence, i.e. a measure of response amplitude, with phase, i.e. a measure of which stimuli elicited the strongest response. The phase-specified coherence values range between −100% and 100%; positive values reflect stronger responses to the 1.5 Hz stimulus presentations, whereas negative values reflect stronger responses to the 7.5 Hz stimulus presentations.

To estimate *φ*_*hrf*_ (or reference phase), we ran a clustering algorithm (k-means) to the phase-coherence data in V2. The number of clusters was set to 2. Starting values of the 2 centers were forced to be the in the first and third quadrants of the phase-coherence data, by computing the median phase and the median coherence values of the corresponding quadrants. The slope of the line passing through the 2 centers was taken as the estimated reference phase. The average reference phase between participants was computed and used in the analysis (reference phase, *φ*_*hrf*_ = 0.68 rad).

Activation maps were then obtained by fist collapsing the phase-specified coherence across cortical layers by taking its mean value, and subsequently projecting the mean values on the inflated cortical surface for each subject (Fig. 1). Cortical surfaces were obtained from the corresponding T1w anatomical images per subject (see Anatomical data analysis).

To quantify the stability of the resulting phase-specified coherence maps, we computed the Spearman correlation coefficient between two independent halves of the data, i.e. odd/even runs and datasets collected on different days. The correlation significance was assessed with analysis of variance.

To estimate the spatial periodicity of the activation (stripe-based) patterns for each region of interest (ROI), i.e. V1. V2, V3 in each participant, we computed the geodesic distance over the cortical surface between each voxel in a given ROI and its neighboring voxels up to 5 mm (radius), using the Dijkstra algorithm. On each iteration (for each voxel in the ROI) the geodesic distance itself and the absolute difference between the phase-specified coherence of the selected voxel with respect to the voxels along the distance, were stored in two separate arrays. The so obtained geodesic distance array was divided into 20 bins, based on 5% percentiles (in this way bins were equal in size). The absolute phase-specified coherence values for each bin were computed and averaged across the participants. The relation between the binned geodesic distance and the absolute phase-specified coherence was assessed fitting an inverted Gaussian function, with mean parameter fixed on 0, letting the standard deviation, amplitude and offset parameter to vary by minimizing the residual sum of squares between the observed data and expected function. The inverted Gaussian function fit for each ROI was obtained from 5000 bootstrapped samples (with replacement)^57^. For each bootstrapping iteration, the full-width half-max (FWHM) of the Gaussian function was stored, yielding a bootstrapped distribution of FWHM values from which 95% confidence intervals were computed. The same procedure was applied to T1w intensity along the geodesic distance. The median FWHM parameter value (and it’s 95% confidence interval) is reported as an estimate of periodicity (full cycle) for phase-specified coherence as well as T1w intensity values.

To quantify whether activation patterns in V2 change abruptly at the V1/V2 border, we computed phase-coherence profiles along the cortical surface orthogonal to the V1/V2 border. Profiles included all V1 and V2 phase-coherence values, up to a maximum distance of 10 mm from the border. This representation allows us to examine changes along the V1/V2 border. Coherence profiles along the cortical surface were also computed for comparison, expecting that the overall fMRI response modulation will be similar along the V1/V2 border.

### Anatomical data-analysis

The T1w anatomical MRI data were skull-stripped, and corrected for large length-scale linear intensity gradient along the slice direction using a linear fit, i.e. the data were normalized by the fitted intensity slope along the slice direction. The scans acquired per subject were then averaged using rigid-body co-registration in AFNI. Gray and white matter was segmented from this image using mrVista and hand-edited to minimize segmentation errors. The cortical surface was reconstructed at the white/gray matter border and rendered as a smoothed 3D surface. This surface served as anatomical background to represent the functional data and the myelin-contrast T1w maps, the latter obtained from the same anatomical dataset.

To quantify the distributed T1w intensities across the cortical thickness, we took the median intensity. In other words, T1w maps of myelin content in gray matter were computed by taking the median of the T1w intensity across the cortical thickness and along neighboring nodes of the cortical surface. A median is less sensitive than a mean to outliers, which may arise due to segmentation errors, partial volume effects but also biological signal variations such as the stria of Gennari. This procedure highlights deviations in overall T1w image intensity associated with myelin content throughout the cortical layers.

After collapsing across cortical thickness, a high-pass filter along the cortical surface removed large-scale intensity variation resulting from B1 and B0 inhomogeneities within the T1w map. To do this we first applied heat-kernel kernel smoothing of 25 mm FWHM^58^. Second, we removed this smoothed map from the T1w-intensity map, leaving smaller scale variations in T1w intensity. Because previous postmortem^59^ and *in vivo* MRI studies^36, 38, 60^ have found a significant correlation of myelin density with the folding pattern of the cortex (curvature), we removed the curvature contribution using a procedure similar to Sigalovsky *et al*.^60^.

Similar to the functional data, we quantified the stability of these T1w intensity maps by computing the Spearman correlation coefficient across the data-points within a visual field map (V1, V2, V3) between two independent halves of the data, i.e. odd/even runs and two datasets collected on different days. The correlation significance was assessed with analysis of variance.

### Correlation between functional and anatomical data

Both functional and anatomical data were collapsed across cortical layers by taking the median value, but otherwise identical to the descriptions above. To decrease the influence of outliers, we excluded the 2.5% tails of the distributions (two-sided) of the BOLD signal intensity and of the curvature value computed for the T1w data within each visual field map (excluded 5% total). The results were robust for changes in the exact threshold values. Functional-anatomical correlations for each participant and area of interest were obtained from 5000 bootstrapped samples (with replacement). For each bootstrapping iteration, the high-pass filtered and de-curved T1w-intensity was divided into 20 bins, based on 5% percentiles of the distribution (in this way bins were equated in size). The corresponding median phase-specified coherence values for each bin were computed. The relation between the binned T1w-intensity and phase-specified coherence was assessed fitting a general linear model. The slope between the binned T1w-intensity and phase-specified coherence was stored on each iteration, yielding a bootstrapped distribution of slope values for each area and participant. The slope distribution for each participant was normalized in the interval [−1 1]. Median and 95% confidence interval were obtained from the normalized slope distribution for each area of interest.
